# A loaded self-managed exercise programme for patellofemoral pain: a mixed methods feasibility study

**DOI:** 10.1186/s12891-019-2516-1

**Published:** 2019-03-27

**Authors:** Benjamin E. Smith, Paul Hendrick, Marcus Bateman, Fiona Moffatt, Michael Skovdal Rathleff, James Selfe, Toby O. Smith, Pip Logan

**Affiliations:** 1 Physiotherapy Department (Level 3) London Road Community Hospital, University Hospitals of Derby and Burton NHS Foundation Trust, Derby, DE1 2QY UK; 2 Division of Rehabilitation and Ageing, School of Medicine, University of Nottingham, Nottingham, UK; 3 Division of Physiotherapy and Rehabilitation Sciences, School of Health Sciences, University of Nottingham, Nottingham University Hospitals (City Campus), Nottingham, UK; 4 Research Unit for General Practice in Aalborg, Department of Clinical Medicine, Aalborg University, Aalborg, Denmark; 5 Department of Occupational Therapy and Physiotherapy, Department of Clinical Medicine, Aalborg University Hospital, Aalborg, Denmark; 6 Department of Health Professions, Manchester Metropolitan University, Manchester, UK; 7 Nuffield Department of Orthopaedics, Rheumatology and Musculoskeletal Sciences, University of Oxford, Oxford, UK

**Keywords:** Mixed-methods study, Feasibility, Patellofemoral pain, Anterior knee pain, Exercise therapy

## Abstract

**Background:**

A novel loaded self-managed exercise programme that includes pain education and self-management strategies may result in better outcomes for people with patellofemoral pain (PFP). However, establishing program feasibility is an essential first step before testing efficacy. The purpose of this study was to evaluate the feasibility and acceptability of conducting a definitive RCT which will evaluate the clinical and cost-effectiveness of a loaded self-managed exercise programme for people with PFP compared with usual physiotherapy.

**Methods:**

In a mixed methods, pragmatic, randomised controlled feasibility study, 60 participants with PFP (57% female; mean age 29 years) were recruited from a physiotherapy clinic within a large UK teaching hospital. They were randomly allocated to receive either a loaded self-managed exercise programme (*n* = 30) or usual physiotherapy (n = 30). Feasibility indicators of process, resources, and management were collected through follow-up of standardised questionnaires six months after recruitment and semi-structured interviews with 20 participants and physiotherapists.

**Results:**

Recruitment rate was 5 participants per month; consent rate was 99%; adherence to intervention appointments was 87%; completeness of questionnaire data was 100%; and adherence to intervention delivery was 95%.

Three exercise diaries were returned at six months (5%). At six months, 25 questionnaire booklets were returned (9 in the loaded self-managed group, 16 in the usual physiotherapy group), with a total retention rate of 42%.

At six months, 56% (5/9) of respondents in the loaded self-managed group and 56% (9/16) in the usual physiotherapy group were classified as ‘recovered’.

Both groups demonstrated improvements in average pain (VAS), kinesiophobia, pain catastrophizing, general self-efficacy and EQ-5D-5 L from baseline to six months.

**Conclusion:**

The results of this feasibility study confirm that it is feasible and acceptable to deliver a loaded self-managed exercise programme to adults with PFP in an NHS physiotherapy outpatient setting. However, between group differences in lost to follow up and poor exercise diary completion mean we are uncertain on some feasibility aspects. These methodological issues need addressing prior to conducting a definitive RCT.

**Trial registration:**

ISRCTN 35272486. Registered 19th December 2016.

## Background

Patellofemoral pain (PFP) is one of the most common forms of knee pain in adults under the age of 40 [1–3]. It has an estimated prevalence of 23% in the general population [4]. The long-term prognosis for PFP is poor [5]. Only one-third of patients are pain-free one year after diagnosis [5], and 91% still report pain and dysfunction four years post-diagnosis [6].

There remains scientific debate around the underlying aetiology of the condition [7]. It is thought most likely to be multifactorial in origin [8]. There is currently little high-quality level 1 evidence to base conservative management on [9]*.* Even in relation to exercise therapy, which has the strongest evidence-base [9], there remains insufficient evidence to determine the best form and dose of exercise [10].

Many patients with PFP develop associated pain- related fear, such as fear-avoidance, catastrophizing thoughts and low self-efficacy [11–14]. These psychological factors not only affect function and quality of life of patients with pain but can modulate the individual pain experience. This may influence the development or maintenance of chronic pain states [15–21]. A systematic review of self-management interventions for chronic musculoskeletal (MSK) pain (16 studies; *n* = 4047), found self-efficacy and depression were the strongest prognostic factors for pain and disability (irrespective of the intervention) [22]. Pain catastrophizing and increasing physical activity were the strongest mediating factors [22]. This provides a foundation that interventions should be aimed at improving pain-related fear and increasing physical activity in relation to self-management strategies.

Exercise therapies designed to load and temporarily aggravate patients’ symptoms have demonstrated improvements for a range of MSK disorders including tendon pain [23], shoulder pain [24–26], low back pain [27, 28] and plantar heel pain [29]. The potential rationale and mechanisms behind loaded painful exercises include positive changes to central and peripheral pain mechanisms, the immune system and affective aspects of pain [30]. Specifically, these exercises are prescribed to address pain-related fear within a framework of ‘hurt not equalling harm’, with the pain experience rationalised as a consequence of ‘de-conditioned’ tissue. Hypothetically, over time, the exercises reduce pain related-fear and the overall sensitivity of the central nervous system, with a modified pain experience [30, 31].

Exercise interventions for PFP have shown a ‘dose-response’, where the greater the volume and intensity of exercise the patient performs the greater their improvement in long-term pain and function [32, 33]. A recent systematic review of painful exercises versus pain-free exercises for chronic MSK pain found regimes using painful exercises offered a small, but significant benefit over pain-free exercises in the short-term. It also reported that regimes using painful exercises typically have higher loads and dose of exercise [31]. Nonetheless, the optimal dose of exercise for the greatest improvements in PFP remains uncertain [10].

Based on these uncertainties, high-quality research on exercise prescription in relation to pain mechanisms and response to load/resistance warrants further investigation. However, to ensure the success of a large multi-centred randomised controlled trial (RCT), several feasibility questions needed to be answered.

The primary aim of this study was to establish the feasibility and acceptability of conducting a definitive RCT which will evaluate the clinical and cost-effectiveness of an intervention based on pain science (where exercises are designed to load and temporarily aggravate patients’ symptoms), self-management strategies and improvements in physical activity levels for people with PFP compared to usual physiotherapy. The intervention has been referred to as a loaded self-managed exercise programme.

## Methods

This study was reported in accordance with the Consolidate Standard of Reporting Trials (CONSORT) statement [34] and Template for Intervention Description and Replication guidelines (TiDieR) [35].

The protocol was approved by the West Midlands - Black Country Research Ethics Committee (ref: 16/WM/0414) and sponsored by University Hospitals of Derby and Burton NHS Foundation Trust. A full description of the methods has been previously published [36]. A brief description is detailed below.

### Study design

A pragmatic, randomised controlled, single-centre, feasibility study, with an embedded qualitative component.

### Participants

Participants were recruited between February 2017 and January 2018 from a physiotherapy waiting list at a large NHS teaching hospital. Patients were referred from general practitioners and from orthopaedics and rheumatology hospital departments. An introductory letter accompanied by an information sheet and consent form was sent out to potential trial participants by a member of the clinical team. This was followed up by a telephone call from a member of the clinical team offering further information and enquiring about participation. Inclusion and exclusion criteria followed currently accepted criteria [37] and were checked both verbally (by telephone initially, then face to face by the same physiotherapist with 10 years’ MSK experience) (Table 1). The same physiotherapist took consent, before baseline data was taken and then randomisation.

**Table 1:** Participant eligibility criteria

Inclusion criteria	
• Aged 18 to 40 years • Greater than three months duration • Clinical diagnosis of unilateral or bilateral patellofemoral pain (if bilateral the worst knee was investigated) • Anterior or retropatellar pain reported on at least two of the following activities: prolonged sitting, ascending or descending stairs, squatting, jumping and running	
Exclusion criteria	
• Previous knee surgery or awaiting lower limb surgery • Knee ligamentous instability • History of patellar dislocation • True knee locking or giving way • Reasons to suspect systemic pathology or acute illness • Patellar or iliotibial tract tendinopathy • Pregnancy or breast feeding • Not able to speak or understand English	

### Sample size

Sixty participants were planned to be recruited; 30 participants per group. The qualitative component interviewed a purposive sample of 20 participants; 10 treating physiotherapists (five from each group), and ten patients (five from each group).

A formal sample size calculation was not performed since the study was designed as a feasibility study. Sample sizes between 24 [38] and 50 [39] have been recommended as providing suitable data for performing a sample size calculation. Therefore accounting for an attrition rate of 20%, these sample sizes were chosen for the feasibility RCT to provide sufficiently robust, meaningful amounts of information [40].

### Randomisation

Patients were randomised to either the intervention group (loaded self-managed exercises) or the control group (usual physiotherapy) (1:1) by a web-based randomisation service with secure password protected login using random variable block-size.

Due to the nature of therapeutic interventions, blinding of the participants and physiotherapists was not possible [41], and participants were aware of the purpose of the study. All participants were blinded to the criteria for feasibility.

### Interventions

#### Training of the physiotherapists

The training package was delivered to the treating physiotherapists by the research team. The training package was designed to be easily deliverable and in a short space of time. It consisted of two, two-hour training sessions, scheduled to fit into the department’s usual in-service training slots.

The first session delivered to all physiotherapists consisted of: background to PFP, rationale for further study, overview of research design, clinical equipoise, usual physiotherapy, discussion and, questions and answers.

The second training session, delivered only to physiotherapists delivering the loaded self-managed intervention, consisted of: revision of training session one, pain education, the loaded exercise, self-management strategies, discussion and, questions and answers.

All physiotherapists were supported to continue giving the interventions through weekly informal chats.

### Loaded self-managed exercise programme

The ‘experimental’ intervention was a loaded self- managed exercise programme. It is a novel intervention based on pain science (where a single exercise is designed to load and temporarily aggravate patients’ symptoms), self-management strategies and improvements in physical activity levels, delivered by trained and supported NHS physiotherapists [36]. The intervention was set within a framework of reducing fear-avoidance, with an emphasis on participant self-management of the condition and exercise programme, and improvements in physical activity levels [36]. A full description of the intervention has been previously published [36]. A brief description is detailed below.

Education of the patients regarding pain mechanisms, such as addressing any beliefs or fear within the participant that pain was a sign of tissue damage, was planned to take up a large portion of the clinical time. Patient discourse regarding tissue-based pathology models of pain, e.g. patellar mal-tracking, or limb mal-alignment was actively discouraged by the physiotherapist.

The physiotherapists prescribed the exercise and typically involved body weight resistance in the form of a modification of the ‘Step Down’ function test [42], a single leg squatting exercise sideways on a step. The exercise required balance, knee extension strength, eccentric control and isometric hip strength. The participants were advised to exercise to the point of fatigue, such that it reproduced their pain and discomfort, whilst ensuring pain was manageable [43–45].

Exercise progression was guided by the symptomatic response. Participants were advised that on cessation of the exercise, the pain should remain no worse than pre-exercise [43]. Participants with more severe pain were able to start on a lighter regime, guided by the baseline functional assessment by the treating physiotherapist. Regression of the exercise programme was reduced repetitions or lightening the exercise, for example moving from single-leg squats to double-leg squats or isometric static squats. Progression was in the form of increased repetitions or increasing the load by moving to plyometric exercises, such as jumping and hopping, for younger participants with higher sporting requirements.

Participants were taught one exercise, to aid adherence [46], whilst being time-efficient [47]. Participants were advised to perform the exercise twice daily. They were encouraged to self-direct progressing/regressing the repetitions, as guided by their pain response. This was done to promote internalising the locus of control and towards self-management and overall improvements in physical activity levels [48].

Self-management strategies employed included: goal-setting, discussions about managing ‘flare ups’ and potential or perceived barriers to successful outcomes of the intervention [47, 49].

Keeping the treatment pragmatic, the timing of follow-ups, number of treatment sessions, frequency and discharge, and physiotherapy-led passive treatments was at the discretion of the qualified physiotherapist. However, as the aim of the programme was focused on self-management strategies, self-directed exercises were promoted and concomitant treatments discouraged. All participants had the opportunity to telephone for support if required, as per usual department practice.

To avoid cross-contamination between the two groups, the intervention group was delivered by physiotherapists who were excluded from treating participants from the control group (and vice versa).

### The comparator

The comparator was usual physiotherapy as directed by the clinical judgement of the treating physiotherapist [50]. Usual physiotherapy often involves strengthening exercises, taping, stretches, foot orthoses, movement retraining and is typically aimed at reducing load on the patella and avoidance of painful exercise and activity [9, 50].

### Outcomes

The following outcomes were measured.

## Feasibility outcomes

### Recruitment & eligibility

Recruitment rates were recorded and defined as the number of participants recruited each month, compared with expected and feasible recruitment rates. We had expected to be able to recruit 4.6 patients per month, based on estimates on the referral rate which was observed in the department between January 2013 and October 2013.

The consent rate was calculated by dividing the number of individuals who met inclusion criteria, by the number who consented to participate in the study.

### Randomisation & blinding

Randomisation was assessed on the rate of participants randomised after consent, and on any challenges reported by the recruiting researcher. Baseline demographic data of age, sex and duration of symptoms were collected.

### Adherence & acceptability

Compliance levels with the intervention were monitored through a participant activity diary. Participants were asked to complete an exercise diary daily for six months to indicate how many exercise repetitions they had completed each day. Adherence to treatment was assessed by the adherence rate to treatment (%) from exercise diaries returned at six months, calculated by the percentage of days they indicated they completed their exercise(s). Adherence to appointments (%) was based on the number of ‘did not attend’ (DNAs), where a participant fails to attend their physiotherapy appointment.

### Patient-reported outcome measures

The retention rate / lost at follow-up was assessed on the percentage of returned outcome forms at three and six months. The percentage of missing data was also recorded.

### Resources & study management

Participant processing time was measured as the number of days from initial contact (information letter being sent) to consent and randomisation.

Fidelity was defined as adherent and competent delivery of the intervention, and was evaluated by analysis of the physiotherapists’ clinical notes against a three-point checklist outlining important details and components of intervention to be completed by the physiotherapist. The three-point checklist included: specific pain education; delivery of a loaded exercise programme; and discussion on self-management strategies, which, as previously mentioned, where main topics discussed during the intervention training. This analysis was conducted for both the loaded self-managed, and usual physiotherapy groups, with a full analysis of treatments delivered in the usual physiotherapy group.

### Patient-reported outcome measures

Clinical outcome measures were collected at baseline, three and six months post-randomisation. The follow-up outcome measures were posted to the patients’ home, with a pre-paid enveloped to return.

The primary outcome measure was the global rating of change (GROC) at follow-up, the proportion of participants who had recovered (defined as ‘completely recovered’ or ‘strongly recovered’), measured on a seven-point Likert scale ranging from ‘completely recovered’ to ‘worse than ever’ [33, 51, 52].

Secondary outcome measures included: the visual analogue scale (VAS) for pain, average over the last week [53], the Tampa Scale for Kinesiophobia (TSK) [11, 54], the ‘Pain Catastrophizing Scale’ (PCS) [55], the General Self Efficacy Scale (GSES) [56], and the generic health outcome Euro-QOL using UK dataset (EQ-5D-5 L), which included a general health VAS [57]. Participation in leisure time sport or exercises within a week was also recorded. The occurrence of an adverse event as a result of participation within this study was not expected, and therefore no adverse event data were collected.

Participants who had not returned the questionnaires were telephoned after seven days to encourage them to complete and return these.

### Embedded qualitative interviews

A sample of 20 participants from the cohort were interviewed. Purposive sampling was employed to gain maximum variation in participants. Ten patients were selected based on the population in terms of intervention groups (both the loaded self-managed group and usual physiotherapy group), age and gender. Attempts were made to include patients who failed to return clinical outcome measures and patients who were classed as non-responders to the treatment (based on the clinical outcome measures) in both groups.

Ten physiotherapists (five from each group) were also interviewed. A purposive sample was selected based on certain characteristics to represent a spectrum population in terms of intervention groups, age, sex and length of time qualified.

Interviews were semi-structured and broadly considered the acceptability and feasibility of study design and training package delivered to physiotherapists.

## Data analysis

### Quantitative data

Reflecting a feasibility study design [58], descriptive statistics along with point estimates, confidence intervals (95%), and effect sizes using independent t-tests, were presented for all appropriate clinical outcome measures. Participant characteristics were presented using means, standard deviations and ranges for quantitative variables and counts and proportions for categorical variables. Feasibility outcomes were described using descriptive statistics. Sensitivity analysis of the primary outcome measure, GROC, was carried out, looking at the proportion of participants who had recovered defined as ‘completely recovered’, ‘strongly recovered’ or ‘slightly recovered’.

Statistical analysis was undertaken using SPSS version 24.0 (Armonk, NY: IBM Corp). No data imputation was performed to account for missing data; intention-to-treat with complete-case analysis was conducted. As recommended by the CONSORT statement, statistical comparison of baseline data was not performed [59].

Feasibility thresholds, as agreed a priori [36], were set at 75% to assess reliability and completeness of outcome measures and used to indicate either success or if strategies are required to improve the viability of any future definite trial, these are presented in Table 2, with feasibility results summarised in Table 3. Where it was not possible to use quantitative data to demonstrate success, outcomes were reported narratively.

**Table 2:** Thresholds for feasibility outcomes

Outcome	Indicator	Successful
Recruitment & eligibility	Recruitment rate (participants per month)	> 3.75
	Consent rate (%)	> 75
Adherence & acceptability	Adherence to appointments (%)	> 75
Outcome measures	Retention rate (%)	> 75
	Completeness of data (%)	> 75
Resources & study management	Adherence to intervention delivery (%)	> 75

**Table 3:** Thresholds for feasibility outcomes - results

Outcome	Indicator	Successful	Result	Feasible	Suggested modifications
Recruitment & eligibility	Recruitment rate (participants per month)	> 3.75	5.0	Yes	
	Consent rate (%)	> 75	98.6	Yes	
Adherence & acceptability	Adherence to appointments (%)	> 75	86.8	Yes	
Outcome measures	Retention rate (%)	> 75	41.7	No	Reduce the number of outcome measures, use of IT (e.g. text message), improve communication between treating physiotherapist and participant, relax criteria for success, entry into a prize draw, telephone consultations.
	Completeness of data (%)	> 75	99.7	Yes	
Resources & study management	Adherence to intervention delivery (%)	> 75	94.9	Yes	

### Qualitative data

The qualitative component followed a thematic analysis approach, as described by Braun and Clarke (2006) [60]. Full details on the qualitative analysis used in this study have previously been published [14].

## Results

### Feasibility outcomes

#### Recruitment & eligibility

**Fig. 1:**
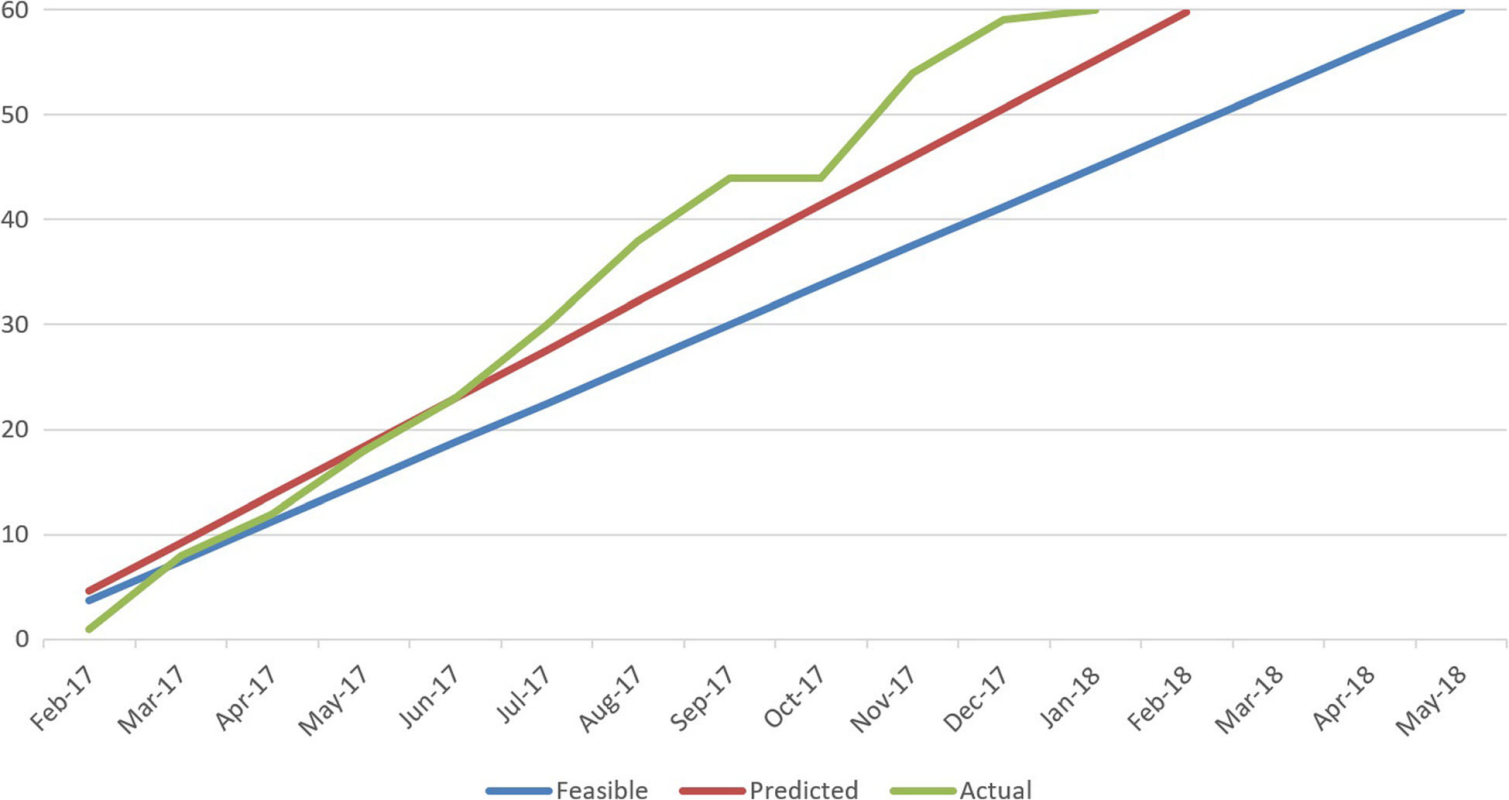
Actual recruitment rate, compared to feasible and predicted

**Fig. 2:**
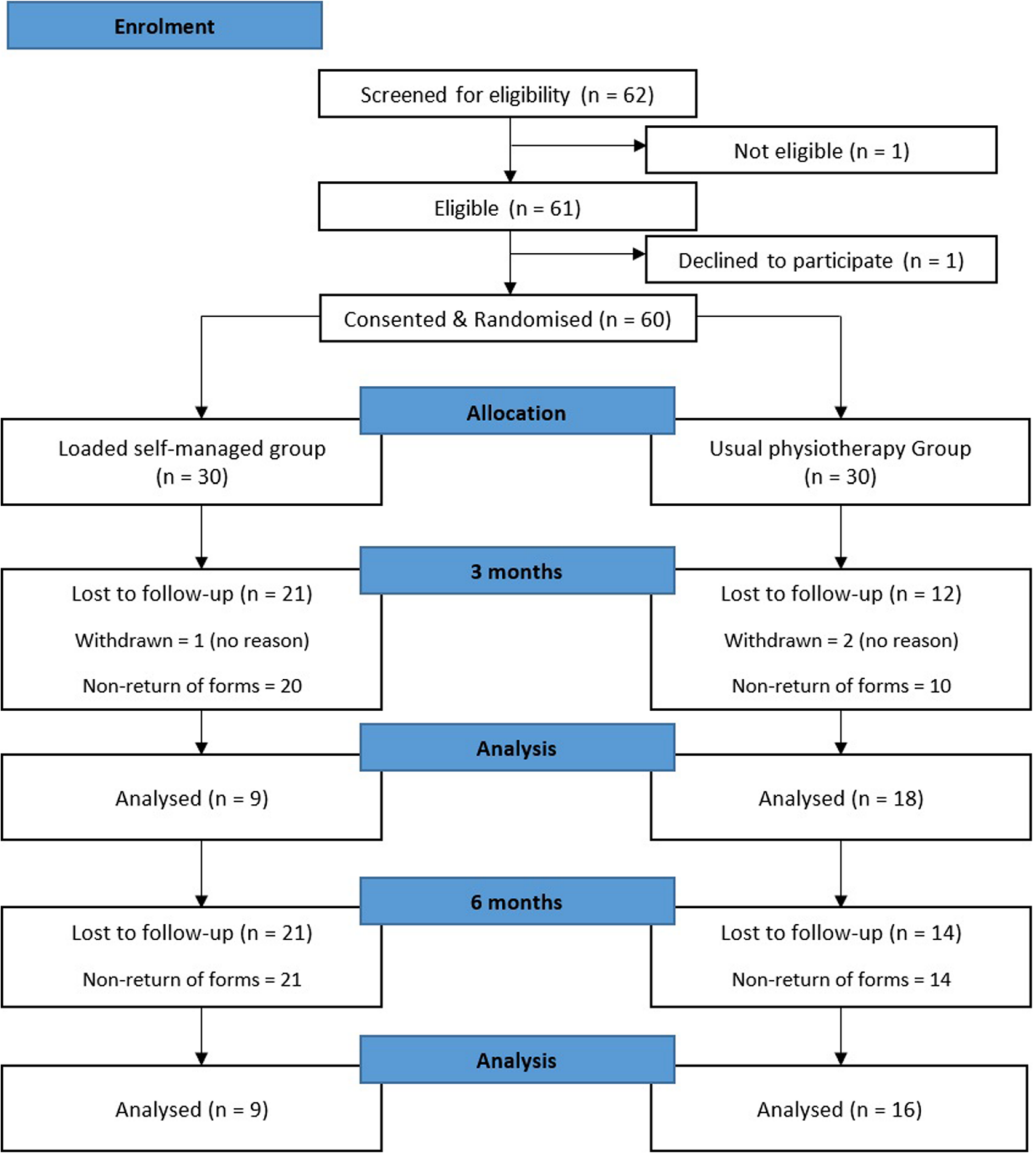
Flow of participants through the study

Recruitment rate was 5.0 participants per month over a 12-month period, exceeding the recruitment rates for feasibility. See Fig. 1 for recruitment rate comparisons, and Fig. 2 for the flow of participants through the study. Over 12-months, 185 referrals were reviewed as potentially eligible, 185 recruitment packs were posted and five (3%) ‘opt out’ slips were returned.

#### Randomisation & blinding

The recruiting researcher reported no randomisation issues. All consenting participants were randomised. There were no baseline imbalances for demographics, baseline symptoms and clinical outcome measures (Table 4).

**Table 4:** Baseline characteristics. Values are means (SD) unless stated otherwise

Characteristics	LSM Group (*n* = 30)	UP (*n* = 30)
Age (years)	31.4 (7.1)	27.4 (6.6)
No of females (%)	15 (50%)	19 (63%)
Duration of knee pain (months)*	18 (6.5–48)	12 (5–27)
Average pain VAS	4.9 (1.9)	4.9 (2.1)
TSK	40.8 (6.5)	37.8 (7.1)
PCS	22.7 (14.1)	19.6 (9.3)
GSES	30.6 (3.2)	31.5 (3.4)
EQ-5D-5 L*	0.65 (0.46–0.72)	0.72 (0.62–0.77)
Health VAS	60.2 (18.4)	72.7 (18.8)
Sport Participation*	2.5 (1–5)	2 (0.0–4)

*Median (interquartile range)

*LSM* loaded self-managed, *UP* usual physiotherapy, *TSK* Tampa Scale for Kinesiophobia, *PCS* Pain Catastrophizing Scale, *GSES* General Self Efficacy Scale, *EQ-5D-5 L* EuroQol 5-dimensions, *VAS* visual analogue scale

#### Adherence & acceptability

Only one exercise diary (3%) was returned from participants in the loaded self-managed group. This indicated exercise adherence of 40% of the time. Two exercise diaries from participants in the usual physiotherapy group (7%) were returned, with a mean adherence rate of 43%.

Adherence to appointments was 87%; with 79% in the loaded self-managed exercise group and 92% in the usual physiotherapy group. The treatments provided by the treating physiotherapists can be seen in Table 5.

**Table 5:** Treatments offered by physiotherapists

Possible physiotherapy treatments	Treatments offered by treating physiotherapist	
	Loaded Self-Managed	Usual Physiotherapy
Specific pain education*	23	0
Loaded exercise*	26	0
Self-management strategies*	25	0
Close chain exercise	21	27
General advice	21	20
Open chain exercise	1	23
Orthotics	1	0
Movement re-training	0	11
Hip specific exercise	0	9
Stretches	0	3
VM exercises	0	2
Referral on	0	1
Electrotherapy	0	0
Acupuncture	0	0
Patella taping	0	0
Massage	0	0
Mobilisations	0	0
Other	0	0

*, three-point checklist outlining important details and components of the intervention; VM, vastus medialis

#### Outcome measures

At three month follow-up, participants who had not returned questionnaires were telephoned and reminded. At this stage, one participant (3%) from the loaded self-managed exercise group withdrew, and two participants (7%) from the usual physiotherapy group withdrew.

Differences in lost to follow-up were demonstrated with the return rate of participants’ questionnaire booklets. At three months, 27 questionnaire booklets were returned (nine in the loaded self-managed group, 18 in usual physiotherapy), with a total retention rate of 45%. At six months, 25 questionnaire booklets were returned (nine in the loaded self- managed group, 16 in usual physiotherapy), with a total retention rate of 42%.

Of the returned forms, all were completed fully, apart from one participant omitting the Tampa Scale for Kinesiophobia at three months, with completeness of data indicators of 99.7%.

#### Resources & study management

The mean participant processing time, from the initial date the research team sent out the recruitment pack to date of participant consent, was 18 days.

The mean number of physiotherapy appointments was 2.4 for the loaded self-managed group, over a mean duration of 3.1 months, compared with 3.2 appointments over 3.8 months for the comparator group.

Fidelity rate in the loaded self-managed group, measured by the three-point checklist was 95% (Table 5). Measuring for contamination, this checklist recorded 0% in the usual physiotherapy group.

#### Patient-reported outcome measures

At three months follow-up, 44% (4/9) of respondents in the loaded self-managed group and 39% (7/18) in the usual physiotherapy group were classified as recovered. At six months, 56% (5/9) of respondents in the loaded self-managed group and 56% (9/16) in the usual physiotherapy group were classified as recovered.

Both groups demonstrated improvements in average pain (VAS), kinesiophobia, pain catastrophizing, general self-efficacy and EQ-5D-5 L from baseline to six months (see Table 6).

**Table 6:** Clinical outcomes. Mean (SD) unless otherwise stated

Outcome	Group	Baseline (SD)	3 months (SD)	6 months (SD)	Mean difference (LSM-UP, 6 months) (95% CI)	ES (*d*) (LSM-UP, 6 months)
Average pain VAS	LSM	4.8 (1.9)	2.9 (1.4)	2.1 (2.2)	−1.2 (− 3.4, 1.1)	0.43
	UP	4.8 (2.1)	3.0 (2.8)	2.4 (2.6)		
TSK	LSM	40.8 (6.6)	34.7 (6.2)	31.3 (8.8)	− 4.3 (− 10.9, 2.3)	0.51
	UP	38.3 (7.1)	34.1 (8.6)	31.6 (6.3)		
PCS	LSM	22.0 (13.8)	16.0 (10.5)	12.6 (9.1)	− 4.0 (− 14.8, 6.8)	0.29
	UP	20.0 (9.4)	16.6 (13.5)	14.1 (10.4)		
GSES	LSM	30.6 (3.2)	29.9 (3.4)	32.3 (2.4)	1.8 (− 1.0, 4.6)	0.53
	UP	31.6 (3.5)	31.2 (3.5)	31.8 (3.2)		
EQ-5D-5 L*	LSM	0.65 (0.53–0.73)	0.75 (0.54–0.82)	0.74 (0.64–0.92)		
	UP	0.71 (0.62–0.79)	0.80 (0.69–1.00)	0.84 (0.73–0.87)		
Health VAS	LSM	60.9 (18.4)	67.8 (16.0)	64.4 (26.4)	8.5 (− 8.1, 25.2)	0.51
	UP	72.1 (19.4)	80.6 (17.0)	79.8 (17.4)		
Sport Participation*	LSM	3.0 (1.0–5.0)	2.0 (1.5–4.0)	3.0 (2.0–6.0)		
	UP	2.0 (0.0–4.0)	3.5 (2.0–4.3)	3.5 (2.0–5.0)		
GROC	LSM		44.4% (4 / 9)	55.6% (5 / 9)		
	UP		38.9% (7 / 18)	56.3% (9 / 16)		
GROC**	LSM		100% (9 / 9)	77.8% (7 / 9)		
	UP		77.8% (14 / 18)	87.5% (14 / 16)		

*LSM* loaded self-managed, *UP* usual physiotherapy, *CI* confidence interval, *TSK* Tampa Scale for Kinesiophobia, *PCS* Pain Catastrophizing Scale, *GSES* General Self Efficacy Scale, *EQ-5D-5 L* EuroQol 5-dimensions, *VAS* visual analogue scale, *GROC* global rating of change scale, *ES* effect size

*, Median (interquartile range); **, sensitivity analysis

## Embedded qualitative interviews

### Recruitment and randomisation

All participants’ comments about the information sheets and letters sent to their home were positive, with no concerns raised.

“Yeah, they really helped me to decide [to take part].”

Also, participants were generally positive about the recruitment process to the feasibility RCT, including receiving a telephone call, the appointment and randomisation process.

### Outcome measures

Participants reflected upon appropriateness, ease of filling in and returning, and the time taken to complete the questionnaire booklets. All, patients were positive about them, with seven patients happy to give no feedback.

“Yeah. The questions, I mean, all seemed fairly normal. I wouldn't have any issues understanding what it was actually asking me or anything.”

Three participants provided feedback about the clinical outcome measures, including recommendation on the addition of a text-box to write open text, if needed; less ambiguity about if the questions were being asked in relation to just their knee pain, or their whole body; wording of the physical activity questions that one patient felt implied they were not already physically active; and use of the word ‘accident’ in a patient group where the pain usually developed insidiously.

Five participants were contacted who had failed to return any outcome measures. All five initially agreed to be interviewed; unfortunately, four failed to attend. Of the ten patients who were interviewed, five returned all outcome measures, four returned one, and one patient failed to return any outcome measures. Of the patients who failed to return all of their outcome measures, four of them stated that they had, suggesting some problem with the pre-paid envelopes and return of the paperwork back to the physiotherapy department at the hospital.

However, the one participant who did acknowledge failing to return one of their outcome measure packs reported that it was due to forgetting, with some difficulty regarding living in different places as a university student.

“Think I might have forgotten it and then ended up leaving it at home before I came. Because I live in halls here.”

### Overall study design

Participants were also asked to reflect on the ways in which study design could be improved. In addition to the feedback already mentioned about the outcome measures, other improvement ideas were: the use of text message reminders (filling in and returning the outcome forms) and information concerning what would happen if they did not respond to treatment whilst in the study.

Finally, physiotherapists were asked to reflect upon, and discuss, their thoughts on the training package delivered at the start of the feasibility RCT. No major concerns or improvements were mentioned; however, the physiotherapists did reference the moderate frequency of other clinical trials running in the department, particularly ones incorporating pain science, suggesting a practised competence at interpreting new information and implementing into clinical practice, that departments not accustomed to clinical trials may find difficult.

“No, it wasn't a culture shock or anything that I felt I needed to kind of do very differently.”

## Discussion

The results of this study confirm that it is feasible and acceptable to deliver a loaded self-managed exercise programme to adults with PFP in an NHS physiotherapy outpatient setting. However, differences in lost to follow up and poor exercise diary completion mean we are uncertain on some feasibility aspects, with potential for systematic bias. Further feasibility work may be needed to address these issues, before supporting a larger clinical trial which will evaluate the clinical and cost-effectiveness of a loaded self-managed exercise programme for people with PFP compared with usual physiotherapy.

### Process

The observed recruitment rate was 5.0 participants per month at the single site over 13 months. We had expected to be able to recruit 4.6 patients per month but deemed a recruitment rate of 3.7 feasible. Our initial recruitment strategy was an estimate based on the referral rate (after full screening by a physiotherapist) of 23 per month (based on our inclusion and exclusion criteria), which was observed in the department between January 2013 and October 2013. However, the observed number of referrals during the study was less than the rate observed in 2013, with 16.8 potentially eligible patients referred each month (before full screening). Therefore, any future definitive trial should consider a lower referral rate than we had initially anticipated.

Of the participants interviewed, both patients and physiotherapists, no barriers to recruitment were identified. However, as the embedded interviews were conducted on recruited participants, it may represent a biased sample. Furthermore, it may be that the participants interviewed felt inhibited to provide negative feedback because they were interviewed by the main researcher. From a management perspective, 50 (27.8%) potential recruits were uncontactable via the telephone numbers supplied by the referring healthcare professional. Therefore, any future trial may need to include strategies in the protocol to cross-check potential eligible participants contact details on referrals with primary care databases.

The qualitative interviews identified no barriers or issues with the delivery of the training package to the physiotherapists. However, positive reference was made to the physiotherapy department’s frequency in participating in clinical trials, suggesting the staff were more willing and able to adapt to the suggested evidence-based training package than, perhaps, a department less experienced in clinical trials.

### Resources

One of the biggest challenges with exercise interventions is treatment adherence and monitoring of adherence. With unsupervised exercises, it is unclear to what degree participants have engaged with the prescribed exercise to obtain any therapeutic benefit. This feasibility study used exercise diaries as a measure of self-reported exercise adherence. The three diaries returned indicated exercise adherence of 42% of the time (40% in the loaded self-managed exercise group compared with 43% in the usual physiotherapy group). However, with such a low return rate, the reliability and validity of the data are limited. Recent systematic reviews have highlighted the lack of validated and reliable self-report measures for unsupervised, exercise-based rehabilitation adherence [61, 62]. Therefore, any future definitive trial may need to consider it unfeasible to monitor adherence through self-reported paper-based measures.

Through qualitative interviews with both patients and physiotherapists, we were able to understand the acceptability of the study design and any potential challenges that may occur with implementation into a large RCT. Patients and physiotherapists perceived some value and benefit from the intervention, which can be seen in the effect sizes of the patient reported outcome measures (Table 6), though the degree and nature of this benefit were variable, with the aforementioned un-even lost to follow up.

### Management

The fidelity assessment highlighted some interesting findings on current practice (Table 5). In contrast to current UK wide physiotherapy treatment, and international best practice guidelines [9, 50], very few physiotherapists provided movement retraining exercises, vastus medialis (VM) exercises, hip-specific exercise or stretches; and no physiotherapists in the usual physiotherapy group offered patella taping, joint mobilisations or orthotics. Further qualitative work trying to understand the nature of and impact of the content of the control group was conducted and published separately. Nonetheless, 95% of patients received the intervention as described in the protocol, indicating intervention fidelity was not an issue in this study.

The overall level of missing data for the returned patient-reported outcome measures was negligible (0.3%). However, the return rate was below the feasibility threshold, with a retention rate of 42%. The telephone and postal reminder for non-returned questionnaires did not improve the response. The researcher also asked treating physiotherapists to prompt participants, should they still be receiving physiotherapy management; this accounted for four receipts of outcome questionnaires, each one being completed in the physiotherapy department during a follow-up appointment. It remains unclear why such a large lost to follow-up occurred. Patient representatives of the trial steering committee approved the completion time of the questionnaires, and this was confirmed by the qualitative interviews of the participants. Of the five patients interviewed who failed to return an outcome questionnaire, four stated they had, suggesting some problem with the pre-paid envelopes. However, internal and external testing of the pre-paid envelopes in the UK and hospital mail system operated correctly. One patient did acknowledge to having failed to return one of their outcome questionnaires saying they simply forgot. This compares to 97 and 96% (at 12 months) in the largest PFP RCT trials to date in the Netherlands and Australia respectively [51, 63]. These trials optimised collection of the main outcome measure by telephone follow-ups and e-mail, rather than relying solely on postal mail.

The retention rate was uneven, with 21 lost to follow-up (70%) in the loaded self-managed group compared with 14 (47%) in the usual physiotherapy group at six months. Of note, is the four participants who failed to return outcome forms at six months, who were still under the care of the physiotherapists and then went on to complete their six month outcome data during their follow-up appointments, were all in the usual physiotherapy group. One possible explanation for the uneven lost to follow-up rate could be the fewer appointments received and the shorter period under physiotherapy management that the loaded self-managed group received compared with the usual physiotherapy group. Ten per cent (3/29) of participants in the loaded self-managed group were still under physiotherapy care at the six month follow-up, compared with 50% (14/28) in the usual physiotherapy group. Lost to follow-up is lower when participants outcome schedule occurs with appointments [64]. Indeed, the mean number of appointments were 2.4 and 3.2 in the loaded self-managed group and usual physiotherapy, respectively; this is considerably lower than the 7.7 appointments seen elsewhere in the UK [65]. A future definitive trial should make modifications to address participant engagement with the study, particularly after they have been discharged from physiotherapy. Strategies could include weekly telephone calls, frequent newsletters, and the use of e-mail or text messaging for measuring adherence or patient report outcome measures.

High participant attrition has been observed in different patient populations, in the same physiotherapy outpatients department, in a large scale RCT (56% at six months) [24]. A high attrition rate does not necessarily mean that large-scale RCTs are unfeasible [66, 67], merely that the sample size calculation and recruitment rate should be adjusted to account for this. Further feasibility work may be warranted to test strategies to improve attrition, including, reducing the burden of the number of outcome measures; use of technology, e.g. text-messaging for reminders or using short outcome measures like pain score or GROC; asking the treating physiotherapists to remind the participants at three months; relaxing the criteria for success; using patient incentives to return forms, e.g. entry into a prize draw; using telephone consultations to complete the questionnaires; or telephoning participants at evenings and weekends [64]. Interestingly, feasibility studies looking at web-based patient questionnaires have found equally low return rates (33% at 24 weeks follow-up), demonstrating the complexity of finding solutions to this problem.

### Strength and limitations

The principle strengths of this research are the comprehensive use of a mixed-methods approach, which was based on the recommendations of the Medical Research Council for the evaluation of complex interventions [68]; the use of concealed random allocation; and its pragmatic evaluation of physiotherapy assessment and interventions.

Study limitations included, firstly, the research being conducted at a single centre, thus reducing its generalisability. Whilst baseline demographics, symptom duration and average pain scores were comparable to previous trials in PFP [69, 70]; a definitive RCT would be required to be multicentred to improve generalisability [64]. Secondly, baseline patient-reported outcome measures were completed in front of an unblinded assessor. Future trials should introduce blinded assessors to reduce the risk of bias [64].

As briefly mentioned above, and despite efforts to the contrary, the embedded qualitative component was likely to have produced a biased sample of participants who had largely positive feelings towards the design of the study. Furthermore, they knew they were being interviewed by the main researcher and therefore may have felt obliged to answer questions favourably. Strategies to reduce this risk such as conducting interviews with an independent researcher or collecting anonymised questionnaires may be considered.

Lastly, as the exercise intervention was considered low risk, being commonly used in musculoskeletal pain populations, no adverse event data were collected.

## Conclusion

A loaded self-managed exercise programme designed around: pain education; a loaded exercise programme; and self-management strategies, is feasible and acceptable to deliver in an NHS physiotherapy outpatient setting. However, the present study demonstrates that, even with the appropriate physiotherapist training package, further feasibility work may be needed to address differences in lost to follow up and poor diary completion. These methodological issues need addressing, before supporting a larger clinical trial which will evaluate the clinical and cost-effectiveness of a loaded self-managed exercise programme for people with PFP compared with usual physiotherapy.

## Abbreviations

CI: Confidence interval
CONSORT: Consolidate Standard of Reporting Trials
DNA: Did not attend
EQ-5D-5 L: Euro-QOL
ES: Cohen’s effect size
GROC: Global rating of change
GSES: General self-efficacy scale
HEE: Health Education England
LSM: Loaded self-managed
MSK: Musculoskeletal
NHS: National Health Service
NIHR: National Institute for Health Research
PCS: Pain catastrophising scale
PFP: Patellofemoral pain
RCT: Randomised controlled trial
SD: Standard deviation
TiDieR: Template for Intervention Description and Replication guidelines
TSK: Tampa Scale for Kinesiophobia
UP: Usual physiotherapy
VAS: Visual analogue scale
VM: Vastus medialis
